# Supplementary material for: Capturing Information About Multiple Sclerosis Comorbidity Using Clinical Interviews and Administrative Records: Do the Data Sources Agree?
Source: Healthcare (Basel). 2025 May 28;13(11):1281. doi: 10.3390/healthcare13111281 (PMC12154387; doi:10.3390/healthcare13111281)
Supplement: Supplementary file 1 [file healthcare-13-01281-s001.zip › healthcare-3551296-supplementary.pdf]

**Supplementary_CRF**

## Comorbidity assessment from medical records

Tax code ____________________

Date of enrolment___/___/_____

**Demographic characteristics**

Date of birth ___/___/_____

Sex: M ☐ F ☐

**Clinical characteristics**

EDSS score (EDSS, Expanded Disability Status Scale) ___/___/

Date of diagnosis ___/___/_____

Date of onset ___/___/_____

Disease course:

RR, relapsing–remitting ☐

SP, secondary-progressive ☐

PP, primary-progressive ☐

**Presence of comorbidities**:

Depression ☐

*Historical diagnosis of depression or treatment by physician*

Anxiety ☐

*Historical diagnosis of anxiety or treatment by physician*

Diabetes ☐

*History of diabetes diagnosed and/or treated by physician*

Hypertension ☐

*Diagnosis and/or treatment of hypertension by physician*

Autoimmune disease ☐

*Historical diagnosis of Rheumatoid arthritis, systemic lupus, systemic sclerosis, Sjogren syndrome, ankylosing spondylitis, myasthenia gravis, Hashimoto's thyroiditis, autoimmune haemolytic anemia, psoriasis and psoriatic arthritis*

Chronic lung disease □

*Historical diagnosis of COPD,chronic bronchitis, chronic asthma and respiratory disease*

Hyperlipidemia □

*Historical diagnosis of hyperlipidemia or treatment by physician*

**Supplementary_diagnoses_codes**

Table S1. Diagnoses and specific codes

| **Classes of comorbidities for MS protocol** | **Comorbidity reported in BDA** | **ESE CODE** (payment exemption code) | **ICD-9-CM** (inpatient diagnosis/procedures) | **ATC codes** (outpatient drug delivery) | **SER CODE** (health services) | **Notes** |
|---|---|---|---|---|---|---|
| Diabetes | Type 1 diabetes | 013.250 | 295, 250.01, 250.03, 250.11, 250.13, 250.21, 250.23, 250.31, 250.33, 285, 250.41, 250.43, 250.51, 250.53, 250.61, 250.63, 250.71, 250.73, | A10A*, N03AX16 or N03AX12 | 1433, 1434, 1475, 96591, 96592, 96593, 96594, 96595, 96596 | • exemption granted within the last 10 years (starting from age >= 35)<br>• hospital discharge occurred within the last 5 years<br>• drug prescription dispensed in the last year; A10A* (DDD>50%) and |

| | | | | | | |
|---|---|---|---|---|---|---|
| | | | 250.81, 250.83, 250.91, 250.93 | | | N03AX16 or N03AX12 (DDD>30%)<br>• health service dispensed in the last year |
| Diabetes | Type 2 diabetes | 013.250 | 294, 250.00, 250.02, 250.10, 250.12, 250.20, 250.22, 250.30, 250.32, 285, 250.42, 250.40, 250.50, 250.52, 250.60, 250.62, 250.70, 250.72, 250.80, 250.82, 250.90, 250.92 | A10A*, N03AX16 or N03AX12 | 1433, 1434, 1475, 96591, 96592, 96593, 96594, 96595, 96596 | • exemption granted within the last 10 years (starting from age >= 35)<br>• hospital discharge occurred within the last 5 years<br>• drug prescription dispensed in the last year; A10A* (DDD>50%), N03AX16 or N03AX12 (DDD>30%) |

| | | | | | | |
|---|---|---|---|---|---|---|
| | | | | | | • health service dispensed in the last year |
| Hypertension | Arterial hypertension | 031.401 or 031.405 or 0A31 | 134, 401*, 403*, 405* | C02AC01, C02CA04, C03*, C07*, C08C*, C09* | | • exemption granted within the last 10 years<br>• hospital discharge occurred within the last 5 years<br>• drug prescription dispensed in the last year; C09* (DDD>50%) |
| Chronic lung disease | Asthma | 007.493 | Principal or secondary diagnosis 493* | R03DC03, R03DC01, R03* | | • exemption granted within the last 10 years<br>• hospital discharge occurred within the last 5 years |

| | | | | | |
|---|---|---|---|---|---|
| | | | | | • drug prescription dispensed in the last year ;<br>R03* (DDD>30%); age 45 yrs; prescription dispensed in the last year |
| Chronic lung disease | COPD | 057 | Principal or secondary diagnosis 491*, 492*, 494*, 496*; correction rule 08B 1 | R03* | • exemption granted within the last 10 years<br>• hospital discharge occurred within the last 5 years<br>• age >=45 yrs; prescription dispensed in the last year<br>• R03* (DDD>30%); age 45 yrs; prescription dispensed in the last year |

| | | | | | |
|---|---|---|---|---|---|
| Chronic lung disease | Respiratory failure/oxygen therapy | 024.518.81 | Principal or secondary diagnosis 518.83, 518.84 | V03AN01 | • exemption granted within the last 10 years<br>• hospital discharge occurred within the last 5 years<br>• drug prescription dispensed in the last year (expenditure of at least 400 euros) |
| Autoimmune disease | Rheumatoid arthritis | 006* | Principal and secondary diagnosis 714.0, 714.1, 714.2, 714.30, 714.32, 714.33 | | • exemption granted within the last 10 years<br>• hospital discharge occurred within the last 5 years |

| | | | | |
|---|---|---|---|---|
| Autoimmune disease | Systemic lupus erythematosus | 028* | Principal and secondary diagnosis 710.0 | • exemption granted within the last 10 years<br>• hospital discharge occurred within the last 5 years |
| Autoimmune disease | Systemic sclerosis | 047* | Principal and secondary diagnosis 710.1 | • exemption granted within the last 10 years<br>• hospital discharge occurred within the last 5 years |
| Autoimmune disease | Sjögren syndrome | 030* | Principal and secondary diagnosis 710.2 | • exemption granted within the last 10 years<br>• hospital discharge occurred within the last 5 years |

| | | | | | |
|---|---|---|---|---|---|
| Autoimmune disease | Ankylosing spondylitis | 054* | Principal and secondary diagnosis 720.0 | | • exemption granted within the last 10 years<br>• hospital discharge occurred within the last 5 years |
| Autoimmune disease | Myasthenia gravis | 034.358.0 | Principal and secondary diagnosis 3580* | N07AA02 | • exemption granted within the last 10 years<br>• hospital discharge occurred within the last 5 years<br>• drug prescription dispensed in the last year; N07AA02 (DDD>50%) |

| | | | | | |
|---|---|---|---|---|---|
| Autoimmune disease | Hashimoto's thyroiditis | 056* | Principal and secondary diagnosis 2452 | | • exemption granted within the last 10 years<br>• hospital discharge occurred within the last 5 years |
| Autoimmune disease | Autoimmune hemolytic anemia | 003.283.0 | Principal and secondary diagnosis 2830 | | • exemption granted within the last 10 years<br>• hospital discharge occurred within the last 5 years |
| Autoimmune disease | Psoriasis AND psoriatic arthritis | 045* | Principal and secondary diagnosis 696* | | • exemption granted within the last 10 years<br>• hospital discharge occurred within the last 5 years |

| | | | | | |
|---|---|---|---|---|---|
| Hyperlipidemia | Familial AND non-familial hypercholesterolemia | 025* | Principal or secondary diagnosis 272.0, 272.2, 272.4, 272.9 | C10AA* | • exemption granted within the last 10 years<br>• hospital discharge occurred within the last 5 years<br>• drug prescription dispensed in the last year; C10AA* (DDD>50%) |

ICD-9-CM: International Classification of Disease-9-Clinical Modification; ATC: Anatomic Therapeutic Chemical System; DDD: defined daily dose; * indicates inclusion of all sub-codes related to principal code

**Supplementary_ATC_codes**

Supplementary Table S2. List of active substances and ATC codes used in the *ad hoc* algorithm to identify depression and anxiety in the administrative databases

| | | |
|---|---|---|
| **Anxiety** | Lorazepam | N05BA06 |
| | Alprazolam | N05BA12 |
| | Brotizolam | N05CD09 |
| | Clonazepam | N03AE01 |
| | Chlordiazepoxide | N05BA02 |
| | Flunitrazepam | N05CD03 |
| | Diazepam | N05BA01 |
| | Delorazepam | N05BA49 |
| | Bromazepam | N05BA08 |
| | Prazepam | N05BA11 |
| | Flurazepam | N05CD01 |
| | Triazolam | N05CD05 |
| | Lormetazepam | N05CD06 |
| | Zolpidem | N05CF02 |

**Supplementary_tables_stratifyed**

Table S3a - Agreement between clinical and administrative data for major comorbidities by sex (F, n = 409; M, n=226)

| Comorbidities | Sex | Agreement (%) | κ (95% CI) | BI | PI | PABAK (95% CI) | Positive agreement (%) | Negative agreement (%) |
|---|---|---|---|---|---|---|---|---|
| Depression | F | 64.6% | 0.23 (0.14-0.31) | 0.24 | −0.38 | 0.29 (0.20-0.38) | 43.1% | 74.2% |
| | M | 70.4% | 0.19 (0.08-0.31) | 0.23 | −0.56 | 0.41 (0.29-0.53) | 32.3% | 81.0% |
| Hypertension | F | 88.8% | 0.58 (0.47-0.69) | 0.06 | −0.69 | 0.78 (0.71-0.84) | 64.1% | 93.3% |
| | M | 91.6% | 0.75 (0.65-0.86) | 0.03 | −0.57 | 0.83 (0.76-0.90) | 80.4% | 94.6% |
| Anxiety | F | 84.6% | 0.03 (-0.06-0.12) | 0.10 | −0.83 | 0.69 (0.62-0.76) | 8.7% | 91.6% |
| | M | 88.5% | 0.02 (-0.04-0.006) | 0.10 | −0.88 | 0.77 (0.69-0.85) | 0.0% | 93.9% |
| Autoimmune disease | F | 82.8% | 0.39 (0.24-0.53) | 0.05 | −0.81 | 0.79 (0.73-0.85) | 44.2% | 94.2% |
| | M | 96.0% | 0.38 (0.07-0.70) | 0.02 | −0.93 | 0.92 (0.87-0.97) | 40.0% | 97.9% |
| Hyperlipidemia | F | 90.0 | 0.08 (-0.05-0.22) | 0.05 | -0.89 | 0.80 (0.74-0.86) | 12.8% | 94.7% |
| | M | 93.8% | 0.03 (-0.06-0.02) | 0.01 | −0.94 | 0.88 (0.81-0.94) | 0.0% | 96.8% |
| Chronic lung disease | F | 95.8% | 0.17 (-0.06-0.40) | 0.01 | −0.95 | 0.92 (0.88-0.96) | 19.0% | 97.9% |
| | M | 98.2% | 0.59 (0.23-0.96) | 0.01 | −0.96 | 0.96 (0.93-1.00) | 60.0% | 99.1% |
| Diabetes | F | 99.3% | 0.85 (0.69-1.00) | 0.00 | −0.95 | 0.99 (0.97-1.00) | 85.7% | 99.6% |
| | M | 98.2% | 0.84 (0.68-0.99) | 0.01 | −0.88 | 0.96 (0.93-1.00) | 84.6% | 99.1% |

κ=Cohen's kappa; BI=bias index; PI=prevalence index; PABAK=prevalence-adjusted bias-adjusted kappa, CI=confidence interval

Table S3b - Sensitivity, specificity, positive (+) and negative (−) predictive values for administrative classification of major comorbidities compared with clinical interview (gold standard) by sex

| Comorbidities | Sex | Sensitivity | Specificity | Predictive value (+) | Predictive value (−) |
|---|---|---|---|---|---|
| Depression | F | 70.5% | 63.1% | 31.1% | 90.1% |
| | M | 66.7% | 70.8% | 21.3% | 94.7% |
| Hypertension | F | *53.3% | 97.0% | 80.4% | 89.9% |
| | M | *75.0% | 96.6% | 86.7% | 92.8% |
| Anxiety | F | 5.6% | 96.6% | 20.0% | 87.1% |
| | M | 0.0% | 99.0% | 0.0% | 82.3% |
| Autoimmune disease | F | 34.7% | 96.9% | 60.7% | 91.6% |
| | M | 30.0% | 99.1% | 60.0% | 96.8% |
| Hyperlipidemia | F | 9.1% | 97.1% | 21.4% | 92.4% |
| | M | 0.0% | 97.3% | 0.0% | 96.4% |
| Chronic lung disease | F | *16.7% | 98.2% | 22.2% | 97.5% |
| | M | *75.0% | 98.7% | 50.0% | 99.6% |
| Diabetes | F | 81.8% | 99.8% | 90.0% | 98.8% |
| | M | 78.6% | 99.5% | 91.7% | 98.6% |

*indicates significant difference ($p<0.05$) between sensitivity or positive predictive value by status

Table S4a - Agreement between clinical and administrative data for major comorbidities by age (≤50y, n = 322; >50y, n=313)

| Comorbidities | Age | Agreement (%) | κ (95% CI) | BI | PI | PABAK (95% CI) | Positive agreement (%) | Negative agreement (%) |
|---|---|---|---|---|---|---|---|---|
| Depression | ≤50y | 68.3% | 0.19 (0.09-0.29) | 0.21 | −0.51 | 0.37 (0.26-0.47) | 35.4% | 79.0% |
| | >50y | 64.9% | 0.24 (0.15-0.34) | 0.26 | −0.37 | 0.30 (0.19-0.40) | 43.9% | 74.4% |
| Hypertension | ≤50y | 92.9% | 0.53 (0.36-0.70) | 0.04 | −0.84 | 0.86 (0.80-0.91) | 56.6% | 96.1% |
| | >50y | 86.6% | 0.67 (0.57-0.76) | 0.06 | -0.45 | 0.73 (0.66-0.81) | 75.6% | 90.7% |
| Anxiety | ≤50y | 85.4% | 0.05 (-0.05-0.14) | 0.12 | −0.84 | 0.71 (0.63-0.79) | 7.8% | 92.1% |
| | >50y | 86.6% | 0.01 (-0.10-0.08) | 0.07 | −0.86 | 0.73 (0.66-0.81) | 4.5% | 92.8% |
| Autoimmune disease | ≤50y | 92.6% | 0.42 (0.23-0.61) | 0.04 | −0.86 | 0.85 (0.79-0.91) | 45.5% | 96.0% |
| | >50y | 91.1% | 0.37 (0.19-0.55) | 0.04 | −0.85 | 0.82 (0.76-0.88) | 41.7% | 95.2% |
| Hyperlipidemia | ≤50y | 96.0% | 0.12 (-0.12-0.35) | 0.02 | -0.95 | 0.92 (0.88-0.96) | 13.3% | 97.9% |
| | >50y | 86.6% | 0.02 (-0.10-0.14) | 0.04 | −0.85 | 0.73 (0.66-0.81) | 8.7% | 92.8% |
| Chronic lung disease | ≤50y | 98.1% | 0.39 (0.01-0.78) | 0.01 | −0.97 | 0.96 (0.93-0.99) | 40.0% | 99.1% |
| | >50y | 95.2% | 0.26 (0.01-0.52) | 0.00 | −0.93 | 0.90 (0.86-0.95) | 28.6% | 97.5% |
| Diabetes | ≤50y | 99.4% | 0.83 (0.60-1.00) | 0.00 | −0.96 | 0.99 (0.97-1.00) | 83.3% | 99.7% |
| | >50y | 98.4% | 0.85 (0.72-0.98) | 0.01 | −0.89 | 0.97 (0.94-1.00) | 85.7% | 92.8% |

κ=Cohen's kappa; BI=bias index; PI=prevalence index; PABAK=prevalence-adjusted bias-adjusted kappa, CI=confidence interval

Table S4b - Sensitivity, specificity, positive (+) and negative (−) predictive values for administrative classification of major comorbidities compared with clinical interview (gold standard) by age (≤50y, n = 322; >50y, n=313)

| Comorbidities | Age | Sensitivity | Specificity | Predictive value (+) | Predictive value (−) |
|---|---|---|---|---|---|
| Depression | ≤50y | 62.2% | 69.3% | 24.8% | 91.9% |
| | >50y | 75.4% | 62.5% | 30.9% | 92.0% |
| Hypertension | ≤50y | *45.5% | 98.3% | 75.0% | 94.0% |
| | >50y | *67.7% | 94.9% | 85.5% | 86.9% |
| Anxiety | ≤50y | 4.4% | 98.6% | 33.3% | 86.4% |
| | >50y | 3.0% | 96.4% | 9.1% | 89.4% |
| Autoimmune disease | ≤50y | 35.7% | 98.0% | 62.5% | 94.1% |
| | >50y | 32.3% | 97.5% | 58.8% | 92.9% |
| Hyperlipidemia | ≤50y | 9.1% | 99.0% | 25.0% | 96.9% |
| | >50y | 6.7% | 95.1% | 12.5% | 90.6% |
| Chronic lung disease | ≤50y | 33.3% | 99.4% | 50.0% | 98.7% |
| | >50y | 30.0% | 97.4% | 27.3% | 97.7% |
| Diabetes | ≤50y | 83.3% | 99.7% | 83.3% | 99.7% |
| | >50y | 79.0% | 99.7% | 93.8% | 98.7% |

*indicates significant difference ($p<0.05$) between sensitivity or positive predictive value by status

Table S5a - Agreement between clinical and administrative data for major comorbidities by disability level (EDSS score <3.5, n = 343; ≥3.5, n=292)

| Comorbidities | EDSS score | Agreement (%) | κ (95% CI) | BI | PI | PABAK (95% CI) | Positive agreement (%) | Negative agreement (%) |
|---|---|---|---|---|---|---|---|---|
| Depression | <3.5 | 60.9% | 0.25 (0.16-0.35) | 0.20 | −0.51 | 0.41 (0.32-0.51) | 40.5% | 79.5% |
| | ≥3.5 | 61.6% | 0.19 (0.09-0.28) | 0.27 | −0.36 | 0.23 (0.12-0.35) | 39.8% | 71.9% |
| Hypertension | <3.5 | 91.3% | 0.62 (0.50-0.74) | 0.05 | −0.74 | 0.83 (0.77-0.89) | 66.7% | 95.0% |
| | ≥3.5 | 88.0% | 0.66 (0.56-0.77) | 0.05 | -0.54 | 0.76 (0.69-0.84) | 74.1% | 92.2% |
| Anxiety | <3.5 | 86.9% | 0.05 (-0.05-0.15) | 0.10 | −0.86 | 0.74 (0.67-0.81) | 8.2% | 92.9% |
| | ≥3.5 | 84.9% | 0.01 (-0.10-0.07) | 0.09 | −0.84 | 0.70 (0.62-0.78) | 4.3% | 91.8% |
| Autoimmune disease | <3.5 | 90.1% | 0.44 (0.28-0.59) | 0.06 | −0.81 | 0.80 (0.74-0.87) | 48.5% | 94.5% |
| | ≥3.5 | 93.8% | 0.28 (0.04-0.51) | 0.02 | −0.91 | 0.88 (0.82-0.93) | 48.5% | 94.5% |
| Hyperlipidemia | <3.5 | 94.8% | 0.23 (-0.06-0.46) | 0.02 | -0.93 | 0.90 (0.85-0.94) | 25.0% | 97.3% |
| | ≥3.5 | 87.3% | 0.01 (-0.09-0.02) | 0.04 | −0.87 | 0.75 (0.67-0.82) | 0.0% | 93.2% |
| Chronic lung disease | <3.5 | 97.4% | 0.39 (0.07-0.70) | 0.01 | −0.96 | 0.965(0.91-0.98) | 40.0% | 98.7% |
| | ≥3.5 | 95.9% | 0.23 (0.01-0.51) | 0.01 | −0.95 | 0.92 (0.87-0.96) | 25.0% | 97.9% |
| Diabetes | <3.5 | 98.8% | 0.71 (0.44-0.98) | 0.01 | −0.96 | 0.98 (0.95-1.00) | 71.4% | 99.4% |
| | ≥3.5 | 99.0% | 0.90 (0.80-1.00) | 0.00 | −0.89 | 0.98 (0.95-1.00) | 90.9% | 99.5% |

κ=Cohen's kappa; BI=bias index; PI=prevalence index; PABAK=prevalence-adjusted bias-adjusted kappa, CI=confidence interval

Table S5b - Sensitivity, specificity, positive (+) and negative (−) predictive values for administrative classification of major comorbidities compared with clinical interview (gold standard) by age EDSS score (<3.5, n = 343; ≥3.5, n=292)

| Comorbidities | EDSS score | Sensitivity | Specificity | Predictive value (+) | Predictive value (−) |
|---|---|---|---|---|---|
| Depression | <3.5 | 69.4% | 71.1% | 28.6% | 93.3% |
| | ≥3.5 | 69.8% | 59.8% | 27.8% | 89.9% |
| Hypertension | <3.5 | 55.6% | 97.9% | 83.3% | 92.2% |
| | ≥3.5 | 66.7% | 95.4% | 83.3% | 89.2% |
| Anxiety | <3.5 | 4.8% | 98.3% | 28.6% | 88.1% |
| | ≥3.5 | 2.8% | 96.5% | 10.0% | 87.6% |
| Autoimmune disease | <3.5 | 35.2% | 97.7% | 69.6% | 91.6% |
| | ≥3.5 | 25.0% | 97.8% | 40.0% | 95.7% |
| Hyperlipidemia | <3.5 | *18.8% | 98.5% | *37.5% | 96.1% |
| | ≥3.5 | *0.0% | 95.5% | *0.0% | 91.1% |
| Chronic lung disease | <3.5 | 30.0% | 99.4% | 60.0% | 97.9% |
| | ≥3.5 | 33.3% | 97.2% | 20.0% | 98.6% |
| Diabetes | <3.5 | 62.5% | 99.7% | 83.3% | 99.1% |
| | ≥3.5 | 88.2% | 99.6% | 93.8% | 99.3% |

*indicates significant difference (p<0.05) between sensitivity or positive predictive value by status
